# Effect of Mechanical Surface Treatment on the Repair Bond Strength of the Silorane-based Composite Resin

**DOI:** 10.5681/joddd.2014.011

**Published:** 2014-06-11

**Authors:** Parnian Alizadeh Oskoee, Soodabeh Kimyai, Elham Talatahari, Sahand Rikhtegaran, Fatemeh Pournaghi-Azar, Jafar Sajadi Oskoee

**Affiliations:** ^1^Professor, Department of Operative Dentistry, Faculty of Dentistry, Tabriz University of Medical Sciences, Tabriz, Iran; ^2^Postgraduate Student, Department of Pediatric Dentistry, Faculty of Dentistry, Tabriz University of Medical Sciences; ^3^Assistant Professor, Department of Operative Dentistry, Faculty of Dentistry, Tabriz University of Medical Sciences, Tabriz, Iran

**Keywords:** Air abrasion, Cr: YSGG laser, diamond bur, Er, repair, silorane

## Abstract

***Background and aims.*** A proper bond must be created between the existing composite resin and the new one for successful repair. The aim of this study was to compare the effect of three mechanical surface treatments, using diamond bur, air abrasion, and Er,Cr:YSGG laser, on the repair bond strength of the silorane-based composite resin.

***Materials and methods.*** Sixty cylindrical composite resin specimens (Filtek Silorane) were fabricated and randomly divided into four groups according to surface treatment: group 1 (control group) without any mechanical surface treatment, groups 24 were treated with air abrasion, Er,Cr:YSGG laser, and diamond bur, respectively. In addition, a positive control group was assigned in order to measure the cohesive strength. Silorane bonding agent was used in groups 14 before adding the new composite resin. Then, the specimens were subjected to a shear bond strength test and data was analyzed using one-way ANOVA and post hoc Tukey tests at a significance level of P &0.05. The topographical effects of surface treatments were characterized under a scanning electron microscope.

***Results.*** There were statistically significant differences in the repair bond strength values between groups 1 and 2 and groups 3 and 4 (P &0.001). There were no significant differences between groups 1 and 2 (P = 0.98) and groups 3 and 4 (P= 0.97).

***Conclusion.*** Surface treatment using Er,Cr:YSGG laser and diamond bur were effective in silorane-based composite resin repair.

## Introduction

Tooth-colored composite resin materials have gained wide popularity in recent years. This popularity is attributed to the non-invasive preparation technique and improved adhesion to tooth structures. These materials exhibit predictable long-term stability with annual failure rates that are comparable to composite resin and amalgam in stress-bearing class I and class II cavities.^1^ One of the major drawbacks of traditional composite materials is shrinkage which is an intrinsic property of the resin matrix. Therefore, most manufacturers have focused on increasing the filler load and reducing the proportion of methacrylate resin. However, the shrinkage has remained a major challenge; therefore, changing the properties of the resin matrix seems another pathway to overcome these shortcomings. To achieve this purpose, dental silorane-based composite resins that consist of a new organic matrix (i.e. monomers with a ring-opening oxirane) were marketed.^2^

The long-term stability of dental restorations has improved in recent years, but some main reasons for replacing restorations are small fractures, wear, and chipping.^3,4^ In most cases, intraoral repair of an existing restoration with direct composite resin is preferable to the complete removal of the entire restoration because complete replacement is time-consuming and increases the risk of removing sound tooth structures.^5^

Mostly it has been reported that a proper bond between old composite resin and the newly added direct composite resin can be achieved by a combination of mechanical surface treatment and the use of intermediate bonding agents and silanes, which can enhance repair bond strength.^6,7^ As a result, numerous repair modalities have been evaluated in vitro for conventional methacrylate-based composite resins, such as surface pretreatment with sandblasting, silica coating, silanization, roughening the surface with diamond burs or silicon carbide papers, use of phosphoric acid, different adhesive techniques and preparation methods.^8-12^

It can be assumed that the surface pretreatment of the silorane-based dental composite can be performed similar to the repair of methacrylate-based composite materials, as carried out in a previous study, where the surface was roughened with silicone carbide paper, cleaned with 37% phosphoric acid and coated with the silorane bonding agent;^13^ however, the repair of siloranes has not been yet investigated in detail. Therefore, a need for evaluation of different pretreatment protocols for repair of dental silorane-based composite resin materials is felt.

Recently, the use of pulsed erbium lasers such as Er,Cr:YSGG laser and Er:YAG laser has been described for surface treatments of tooth structures and filling materials.^14^

Silorane-based composite resins and their specific adhesive system are widely available for clinical applications. These restorative systems, whose matrix is composed of organic silorane, are claimed to have lower polymerization shrinkage. This is a result of the silorane chemical reaction, which occurs through a cationic benzene ring-opening procedure, promoting reduced resin shrinkage, when compared with methacrylate-based resins.^15^ Despite improvements in the physical and mechanical properties of composite resins,^16^ fractures and failures still occur.^17^ According to the minimally invasive restorative concept^18^ complete removal of a fractured, stained, or defective complex composite resin restoration is often undesirable.^19^Successful composite resin repair requires an adequate interfacial bond between the old and fresh composite resin.^20-24^ Many surface conditioning methods and adhesion promoters have been proposed to enhance the repair strength of methacrylate-based composite resins.^19,22,24^ Therefore, the aim of the present study was to compare the effect of three mechanical surface treatment modalities, including air abrasion, Er,Cr:YSGG laser and diamond bur, on the repair bond strength of a silorane-based dental composite resin.

## Materials and Methods

In this in vitro study, sixty cylinder-shaped specimens, 4 mm in height and 4 mm in diameter were prepared by layering 2-mm-thick increments of a silorane-based composite resin (Filtek Silorane, 3M ESPE, St. Paul, MN, USA) using plastic molds.

Each increment was cured for 40 seconds with a light-curing unit (Astralis 7, Ivoclar, Vivadent, Lichtenstein) according to manufacturer’s instructions. The last increment was covered with an acetate strip (Hawe Neos Dental, Bioggio, Switzerland) and compressed with a glass slab in order to obtain a smooth surface for specimen after light-curing. Subsequent to the curing of the second layer the specimens were removed from the mold and cured for 20 seconds from each side. Fifteen additional specimens, 6 mm in height and 4 mm in diameter, were prepared in the same manner in order to evaluate the cohesive strength. Then, the composite resin cylinders were embedded in acrylic resins up to a height of 2 mm and were randomly divided in four groups of 15 specimens each.

In group 1 (control group), the specimens did not receive any mechanical surface treatment.

In group 2, the specimen surfaces were air-abraded at a pressure of 60 PSI using an air abrasion device (Microblaster, Dento-prep TM, Dental microblaster, Denmark) for 10 seconds with 50-µm aluminum oxide particles. The tip was positioned 5 mm away from the target and perpendicular to the specimen surface. Subsequently, the specimens were rinsed with distilled water and air-dried.

In group 3, an Er,Cr:YSGG laser unit (Biolase Europe Gmblt, Paintweg 10,92685 Floss, Germany) with a 600-µm diameter, G-type laser tip was used for surface treatment. This laser system emits photons at a wavelength of 2.78 µm, which are pulsed with durations of 140-200 µs and a repetition rate of 20 Hz. The output power of this device can be varied from 0 to 6 W. Laser power of 2 W (100 mJ) at 15% air level and 10% water level was determined to be optimal in the pilot study. The beam was aligned to be perpendicular to the target area and was applied at a 1-mm distance during the exposure time of 5 seconds; subsequently, the specimens were rinsed and air-dried.

In group 4, the surface of specimens was roughened for 3 seconds with a coarse diamond bur (001 cylinder Flat End, SS White Burs, Inc. Lakewood, NJ) placed tangential to the surface and rotating at a high speed with constant water spray. A new bur was used after five bur treatments. Then, the specimens were rinsed and air-dried.

After that in groups 1-4, the bonding agent (Filtek Silorane Bond, 3M ESPE, St. Paul, MN, USA) was used according to manufacturer’s instructions and light-cured for 10 seconds using a light-curing unit (Astralis 7, Ivoclar, Vivadent, Lichtenstein) at a light intensity of 400 mW/cm^2^. Plastic molds (2 mm in height and 3 mm in diameter) were placed over the test surfaces at the center of the samples. Then, Filtek silorane composite resin was applied as a repair material with a thickness of 2 mm and light-cured using Astralis 7 light-curing unit for 40 seconds at a light intensity of 400 mW/cm^2^. Following mold removal, the newly added direct composite resin was light-cured for 20 seconds from each side. The specimens were individually stored in distilled water at 37°Cfor 24 hours.

Then, the specimens were subjected to the shear bond strength test using a universal testing machine (Hounsfield Test Equipment, Model H5K-S, England). A crosshead speed of 1 mm/min was used to load the repaired surface interface. The force was applied by the chisel-shaped blade of the equipment at the interface of the old and new composite resin. The same technique was used to measure the cohesive strength of the samples. Prior to adding new composite resin, two samples from each group were randomly selected and were gold-sputtered by a 150-Å thin gold layer using gold sputtering (Emitech K550, UK) and a vacuum machine (EDWARDS RV5, A 653-01-903, 10^-3^ mbr, Ideal Vacuum Products, LLC); then, the surface topography was evaluated under a scanning electron microscope (Tescan Vega-II; Tescan, S.RO. Libusinia Trida, CZ) at ×200 and kV=10. Shear bond strengths were recorded in Newton and converted to MPa.

Data was analyzed using one-way ANOVA and post hoc Tukey tests. Statistical significance was defined at P < 0.05.

## Results

**Figure 1.**
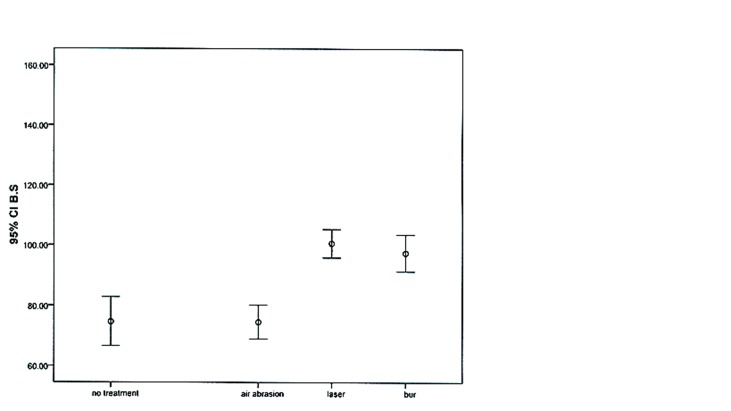
Error bar graph of the repair bond strengths in MPa for the study groups is presented.

Means and standard deviations and error bar graphs of the repair shear bond strength values for the study groups are shown in Table 1 and Figure 1.

**Table 1:** Means and standard deviations of the repair bond strengths in MPa for the study groups

Group	N	Mean	Std. Deviation	Min	Max
Control	15	10.56 A	2.06	7.41	13.84 a
Air abrasion	15	10.55 A	1.43	7.43	13.23 a
Er,Cr:YSGG	15	14.23 B	1.20	12.73	16.27 b
Diamond bur	15	13.78 B	1.56	11.64	16.70 b
Cohesive	15	19.15	3.09	14.89	27.74
Total	75	13.65	3.71	7.41	27.74
Different capital letters indicate that there were no significant differences in the repair bond strength values between groups 1 and 2 (P = 0.98) and groups 3 and 4 (P = 0.97).					
Different lowercase letters indicate significant differences in the repair bond strength values between groups 1 and 2 and groups 3 and 4 (P < 0.001).					

The results of one-way ANOVA demonstrated that statistically significant differences in bond strengths between the study groups (P < 0.001). Two-by-two comparisons of the groups with post hoc Tukey tests revealed significant differences in bond strength between groups 1and 2 and groups 3 and 4 (P < 0.001); however, there was no significant differences in bond strengths between groups 1 and 2 (P = 0.98) and groups 3 and 4 (P = 0.97).

**Figure 2.**
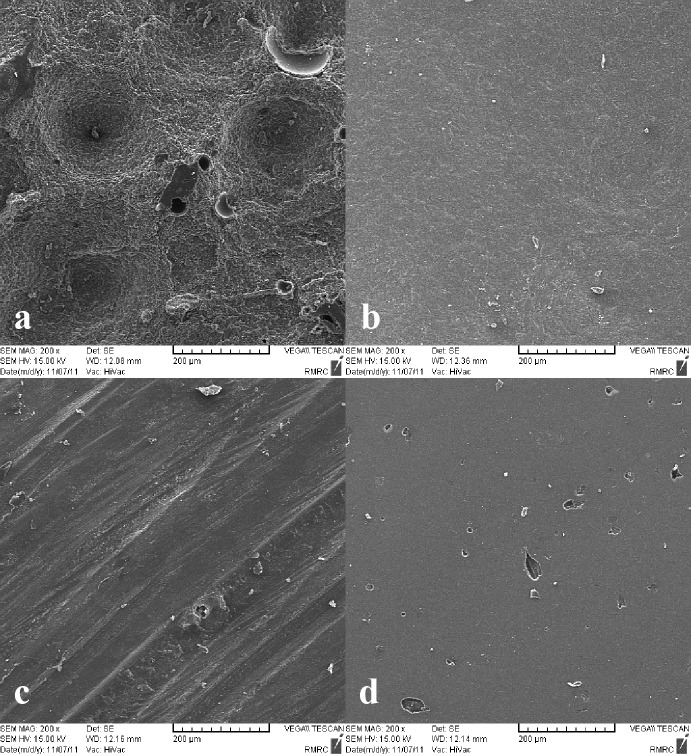
The micrographs of surface topographies of laser-treated (a), air abraded (b), diamond bur-ground (c) and control (d) groups of the silorane-based specimens.

The micrographs of surface topographies of the four study groups are presented in Figure 2. In the Er, Cr: YSGG laser group, a clearly visible and homogeneous micro-retentive feature was seen in the form of surface depressions. Diamond bur prepared the surface in a linear pattern. On the other hand, both the control and air-abraded groups had relatively smooth surfaces.

## Discussion

According to the minimally invasive restorative concept^18^ complete removal of a fractured, stained, or defective complex composite restoration is often undesirable.^19^Improving the bond strength between new and old composite resin restorations usually requires increased surface roughness to promote mechanical interlocking because chemical bonding might not be adequate. Increasing the surface roughness provides better mechanical interlocking and increases the probability of finding residual free carbon bonds through the layer surface area.^2^ The aim of this study was to determine the effect of mechanical surface treatments on the repair shear bond strength of silorane-based composite resins.

The results revealed that surface treatment with Er,Cr:YSGG laser increased the repair bond strength, consistent with the findings of Kimyai et al,^14^ who found that Er,Cr:YSGG laser irradiation is the best surface treatment method for repair of laboratory composite resins. In addition, Navimipour et al demonstrated that the surface treatment of resin-modified glass-ionomer with Er,Cr:YSGG laser increased the bond strength of composite resin to the surface of the resin-modified glass-ionomer.^25^Studies have evaluated the effects of Er:YAG laser groups on ablation of composite resins,^26,27^and have reported that an explosive vaporization is followed by hydrodynamic ejection. During this process, rapid melting and as a result, a change in the volume of the molten material produces strong expansion forces. Interactions between the forces created and the composite resin structure produce projections on the surface and droplets are formed as a result of molten material removal.^27^ It has been suggested that this type of effects takes place in the composite resin ablation subsequent to Er,Cr:YSGG laser irradiation.^28^Electron microscope images in the present study revealed that Er,Cr:YSGG laser irradiation on composite resin resulted in formation of a pitting irregular surface, without smear layer formation, which increases the bonding surface area and better distribution of stresses at the interface,^29^ causing an increase in repair bond strength.

Similar to the Er,Cr:YSGG laser group, the repair bond strength values in the diamond bur group were high and significantly different from the control and air-abraded groups, consistent with the finding of Tabatabaei et al,^30^ who found that diamond bur is the most effective surface treatment for repair of aged composite resin; however, it should be pointed out that laser treatment was not applied in that study. In addition, Brosh et al^19^ found that diamond bur was more effective than sandblasting in surface treatment of water-aged composite resin restorations. Likewise, Bonstein et al^31^ found that surface treatment with diamond burs resulted in higher bond strength compared with air abrasion in the repair procedure of direct composite resins. Diamond bur roughening may create microretentive features in a linear pattern seen under SEM that can increase micro-retention.

Furthermore, in the present study the repair bond strength in air abrasion group was almost similar to that in the control group with statistically significant differences compared to the Er,Cr:YSGG laser and diamond bur groups. Some investigations have demonstrated a reduction in repair bond strength after surface abrasion.^30,32-34^ They have generally attributed this reduction to the exposure of filler particles following abrasion, and hence reduced availability for primary bonding to the resin.^30^Other possibilities are intervention of the surface debris with the repair and the inclusion of air at the interface reducing the surface area available for bonding. Also, the specimen surface topography showed relatively smoother surface in air-abraded group compared to Er,Cr:YSGG laser and diamond bur groups. In contrast, Covalcanti et al^35^reported that surface treatment of direct composite with air abrasion led to higher repair bond strength values compared with diamond burs. Bouschlicher et al^36^ did not find significant differences between the values of repair bond strength while using air abrasion or diamond burs. It has been suggested that various mechanical surface treatments may lead to differences in smearing and matrix cracking that may affect bond strength.^37^ The difference in the repair bond strength values of composite resins might be attributed to differences in treatment protocols, aging period durations, curing methods and the type of composite resin. As a result, the outcomes of each study should be interpreted according to the protocols exercised.

It should be noted that in the present study the mean repair bond strength values in Er,Cr:YSGG laser and diamond bur groups were approximately 70% of the cohesive strength of Filtek silorane composite resin, which is clinically acceptable based on a study carried out by Boyer et al.^24^

Finally, it must be pointed out that composite restorations are highly exposed to the effects of pH changes,^38^salivary enzymes,^39^ and the wet environment,^40-42^which can cause degradation over time. Since aging alters the composition of the material and may influence the repair bond strength,^43^ future studies should pay special attention to investigate the effect of different surface treatments on repair bond strength of silorane-based composites undergoing aging process.

## Conclusion

Within the limitations of this in vitro study, it can be concluded that surface roughening either with Er,Cr:YSGG laser or diamond bur followed by the application of the silorane bonding agent were effective in surface treatment of silorane-based composite resins prior to repair.
